# The diagnosis of tuberculous meningitis in adults and adolescents: protocol for a systematic review and individual patient data meta-analysis to inform a multivariable prediction model

**DOI:** 10.12688/wellcomeopenres.15056.3

**Published:** 2021-01-04

**Authors:** Tom Boyles, Anna Stadelman, Jayne P. Ellis, Fiona V. Cresswell, Vittoria Lutje, Sean Wasserman, Nicki Tiffin, Robert Wilkinson

**Affiliations:** 1 Wits Reproductive Health and HIV Institute, University of the Witwatersrand, Johannesburg, Gauteng, 2001, South Africa; 2 Faculty of Infectious and Tropical Diseases, London School of Hygiene and Tropical Medicine, London, WC1E 7HT, UK; 3 School of Public Health, University of Minnesota, Minneapolis, Minnesota, USA; 4 Hospital for Tropical Diseases, University College London Hospitals NHS Foundation Trust, London, UK; 5 Clinical Research Department, London School of Hygiene and Tropical Medicine, London, WC1E 7HT, UK; 6 Research Department, Infectious Diseases Institute, Kampala, Uganda; 7 Cochrane Infectious Diseases Group, University of Liverpool, Liverpool, UK; 8 Wellcome Centre for Infectious Disease Research in Africa, Institute of Infectious Diseases and Molecular Medicine, University of Cape Town, South Africa; 9 Division of Computational Biology, Integrative Biomedical Sciences, University of Cape Town, University of Cape, South Africa; 10 Department of Medicine, Imperial College London, London, UK; 11 The Francis Crick Institute, London, UK

**Keywords:** Tuberculous meningitis, multivariable prediction rule, machine learning, diagnostics

## Abstract

**Background: **Tuberculous meningitis (TBM) is the most lethal and disabling form of tuberculosis. Delayed diagnosis and treatment, which is a risk factor for poor outcome, is caused in part by lack of availability of diagnostic tests that are both rapid and accurate. Several attempts have been made to develop clinical scoring systems to fill this gap, but none have performed sufficiently well to be broadly implemented. We aim to identify and validate a set of clinical predictors that accurately classify TBM using individual patient data (IPD) from published studies.

**Methods: **We will perform a systematic review and obtain IPD from studies published from the year 1990 which undertook diagnostic testing for TBM in adolescents or adults using at least one of, microscopy for acid-fast bacilli, commercial nucleic acid amplification test for *Mycobacterium tuberculosis* or mycobacterial culture of cerebrospinal fluid.  Clinical data that have previously been shown to be associated with TBM, and can inform the final diagnosis, will be requested. The data-set will be divided into training and test/validation data-sets for model building. A predictive logistic model will be built using a training set with patients with definite TBM and no TBM. Should it be warranted, factor analysis may be employed, depending on evidence for multicollinearity or the case for including latent variables in the model.

**Discussion: **We will systematically identify and extract key clinical parameters associated with TBM from published studies and use a ‘big data’ approach to develop and validate a clinical prediction model with enhanced generalisability. The final model will be made available through a smartphone application. Further work will be external validation of the model and test of efficacy in a randomised controlled trial.

## Introduction

Tuberculosis remains a major global health problem, with the most lethal and disabling form being tuberculous meningitis (TBM), of which there are more than 100,000 new cases each year ^1^. Mortality is high, particularly in children and patients who are co-infected with HIV-1 ^2^. The diagnosis is often delayed by the insensitive and lengthy culture technique required for disease confirmation, with delayed diagnosis and treatment being important risk factors for poor outcome ^1^. Recently introduced nucleic acid amplification tests (NAATs) allow more rapid detection of TBM. Pooled specificity of 98.0% and 90% for Xpert MTB/RIF and Xpert MTB/RIF Ultra respectively, suggest that they are effective rule-in tests with the potential to speed up diagnosis and reduce unnecessary treatments for alternative conditions in some patients. However, the pooled sensitivity is 71.1% and 90% respectively, which is even lower for patients with HIV (58% to 81%) ^3^. Given the extremely high mortality if treatment is withheld from patients with TBM, these values are unlikely to be sufficient evidence to withhold treatment when negative in most patients. Improved strategies to rapidly and accurately diagnose TBM are urgently needed ^1^.

A major stumbling block in TBM research had been the absence of a single reference standard test or standardised diagnostic criteria. In 2010, a committee of 41 international experts in the field developed consensus case definitions for TBM for use in clinical research ^4^. These case definitions have helped to standardise research but are not appropriate for use in routine clinical care as they depend on variables such as cerebrospinal fluid (CSF) culture results, which can take up to 6 weeks to become positive and may include brain imaging, which is not available in many resource constrained settings.

Another approach to improving rapid diagnosis in TBM, particularly in resource-limited settings where the majority of cases occur, is to develop and validate multivariable prediction models. At least 10 models have been published for the diagnosis of TBM, but a major limitation is that their performance is variable in different populations and settings ^1^. A major reason for heterogeneous model performance across different settings and populations is case mix variation, which refers to the distribution of important predictor variables such as HIV status and age, and the prevalence of TBM. Case mix variation across different settings or populations can lead to genuine differences in the performance of a prediction model, even when the true predictor effects are consistent (that is, when the effect of a particular predictor on outcome risk is the same regardless of the study population) ^5^. 

Recent studies have shown how big datasets can be used to examine heterogeneity and improve the predictive performance of a model across different populations, settings, and subgroups ^6– 8^. Individual patient data meta-analysis is preferred to aggregate data meta-analysis, as risk scores can be generated and validated, and multiple individual level factors can be examined in combination ^9^. 

### Objectives

1. Conduct a systematic review to identify studies that applied systematic diagnostic strategies for TBM in adolescents and adults presenting with meningitis
2. Establish an international collaboration among TBM research groups who are willing to provide individual patient data (IPD)
3. Use IPD to develop a clinical prediction model that estimates the probability of TBM in adolescent and adults, based on clinical and laboratory data that is routinely available within 48 hours of initial evaluation

Secondary objectives include an assessment of the number and quality of studies addressing the diagnosis of TBM, as well as an analysis of demographic and clinical characteristics of cases and non-cases of TBM.

## Protocol

A systematic review and IPD meta-analysis will be performed according to Preferred Reporting Items for Systematic review and Meta-Analysis of IPD (PRISMA-IPD) guidelines ^10^.

### Identification of studies

Potentially eligible studies will be identified by an extensive search of electronic databases, manual search of reference lists and by contacting researchers with interest and expertise in meningitis who may have access to unpublished studies.

We have designed a broad search strategy to maximise sensitivity. We will combine medical subject heading (MeSH) and free text terms to identify relevant studies, see Table 1. We will search Medline (accessed via PubMed), Africa-Wide Information and CINAHL (both accessed via EBSCO Host). We will not limit our searches by geographical location. The search will be restricted to studies published after 01 January 1990 and in English. The detailed search strategies will be presented in an online supplementary appendix. Reference lists of the selected articles and reviews will be searched manually to identify additional relevant studies.

**Table 1.** Proposed search terms.

Search	Query
#1	Search tuberculosis meningitis Field: Title/Abstract
#2	Search “tuberculosis, meningeal”[MeSH ]
#3	Search cerebral tuberculosis Field: Title/Abstract
#4	Search “brain tuberculosis” Field: Title/Abstract
#5	Search TBM Field: Title/Abstract
#6	Search ((((tuberculosis meningitis) OR “tuberculosis, meningeal”[MeSH Terms]) OR “cerebral tuberculosis“) OR “brain tuberculosis”) OR TBM
#7	Search “Diagnosis”[Majr]
#8	Search diagnosis or diagnostic Field: Title/Abstract
#9	Search “clinical scores” or “clinical scoring” Field: Title/Abstract
#10	Search “Research Design”[Mesh]
#11	Search predictor* or predictive Filters: Field: Title/Abstract
#12	Search “clinical predict*” Field: Title/Abstract
#13	Search “clinical feature*” Field: Title/Abstract
#14	Search (((#13 OR ((#12) OR ((#11) OR ((#10) OR ((#9) OR #8 OR #7 Filters: Humans
#15	Search #14 AND #6 Filters: Humans

### Types of studies

*Inclusion criteria*

- Randomized controlled trials, cross-sectional studies, and observational cohort studies
- Participants presenting to care with clinical meningitis
- Use of at least 1 of microscopy for acid-fast bacilli, commercial nucleic acid amplification test (NAAT) for *Mycobacterium tuberculosis* or mycobacterial culture of CSF to diagnose TBM
- Study includes a minimum of 10 participants aged ≥ 13 years

*Exclusion criteria*

- Case-control studies and case reports/series of patients with confirmed TBM
- Participants taking anti-TB drugs at the time of their evaluation
- Non-English articles
- Studies published before 1990
- Full text unable to be located
- Studies not in humans

### Screening and study selection

Duplicate studies will be removed. Study selection will follow the process described in the Cochrane Handbook of Systematic Reviews and PRISMA-IPD statements ^10^. Two investigators will independently screen titles and abstracts to remove irrelevant studies. Full text review will be performed on the remaining studies to determine eligibility. Any disagreements will be resolved by consensus or in consultation with a third reviewer.

### Data extraction

Data will be extracted on a proforma, independently by two review authors on study level variables: study setting and dates; contact details; inclusion criteria and exclusion criteria, and number of patients. Corresponding authors of studies identified as eligible after full text review will be contacted with a request to provide anonymised individual patient data. IPD for variables that have previously been shown to be predictive of TBM ^1^ and competing diagnoses will be requested, Table 2. Investigators will be requested to share their anonymised data after obtaining a signed agreement.

**Table 2.** Individual patient data that will be requested from authors. LAM= lipoarabinomannan NAAT= nucleic acid amplification test.

Clinical data at presentation	Laboratory results (blood)	Laboratory results (CSF)
• Age * • Sex * • Presence of extrapyramidal movements * • Presence of neck stiffness * • Duration of symptoms * • Focal neurological deficit (including cranial nerve palsy) * • Temperature * • Glasgow Coma Scale * • AVPU score *	• HIV sero-status * • Total leukocytes * • CD4 count * • Glucose *	• Appearance * • Total leukocytes * • Total neutrophils * • Total lymphocytes * • Protein * • Glucose * • Gram stain * • Adenosine deaminase activity * • Bacterial culture • India ink stain * • Cryptococcal antigen * and culture • Microscopy for acid-fast bacilli • Mycobacterial culture • NAAT for *Mycobacterium tuberculosis* • NAAT for any virus • Syphilis serology * • Any other test informing an alternative diagnosis
Laboratory results (urine, sputum and serous effusions)	Radiological investigations	Autopsy
• Urine LAM * • Microscopy for acid-fast bacilli * • Mycobacterial culture • NAAT for *Mycobacterium tuberculosis* *	• Chest X-ray * • Abdominal ultrasound scan • CT brain • MRI brain	• Histological results from autopsy

*Factors chosen *a priori* to be used to develop the initial model.

### Data management

Investigators will be asked to share anonymised individual patient data, preferably electronically using encrypted files and other secure data transfer technologies using standardised data collection forms. Only study collaborators will have access to the combined IPD data available in Box. Box Secure Storage is a cloud storage and collaboration service configured to meet the security standards for HIPAA data. Data will remain stored in Box for the duration of the study and will not be used or sold for any commercial purpose.

### Authorship

Authors providing IPD will be asked to nominate co-authors to expand the expertise of the review group, including review of preliminary findings and manuscript authorship. The number of co-authors will depend on the amount of data supplied, 1 author for <100 patients, 2 authors for >100 and <250 patients, and 3 authors for >250 patients.

### Quality assessment

Quality assessment in terms of risk of bias and applicability for each included study will be performed according the QUADAS-2 tool for diagnostic accuracy studies ^11^. This tool comprises 4 domains: patient selection, index test, reference standard, and flow and timing. Each domain is assessed in terms of risk of bias, and the first 3 domains are also assessed in terms of concerns regarding applicability. Signalling questions are included to help judge risk of bias.

### Data synthesis

1. Descriptive analysis of available parameters, data completeness check, and IPD meta-analysis.

The contributing datasets will be reviewed for sample size, available variables and data completeness, to inform the selection of a modelling approach. A descriptive analysis will be undertaken to understand similarities and differences between the contributing datasets. Participant characteristics and clinical features ( Table 1) will be summarized for each contributing dataset and compared across datasets using chi-square, t-tests, or non-parametric methods as warranted.

2. Selection of Candidate Predictors

The objective of this step will be to reduce the number of variables that go into the development of the TBM prediction model. Prior studies have indicated several predictive variables such as

- Symptom duration prior to presentation at the hospital (days)
- CSF leukocytes
- CSF neutrophil (%)
- CSF glucose
- CSF protein

We aim to include these variables in the predictive model as “primary predictors” in an effort to retain the variables that are the most predictive of TBM diagnosis as well as easily acquired in low resource settings. Primary predictors will be assessed for missingness, imputed if missing (see Step 3 for more detail), and will be used in predictive model development. Other variables that we would like to include, as “secondary predictors” are,

- Age
- Sex
- Blood glucose
- Blood leukocytes
- Country
- HIV status

We aim to include the above secondary predictors in an effort to explore their predictive value of diagnosing TBM. The secondary predictors have been selected based on prior published diagnostic algorithms. They will be assessed for missingness and imputed if missing (see Step 3 for more detail) but may or may not be included in the final algorithm if their addition to the algorithm does not result in better predictive performance. It is also possible that age, sex, country, and HIV status could explain case-mix variation.

We will also consider employing methods such as principal component analysis and joint individual variation explained ^12^ to identify the variables that explain most of the variation in TBM diagnosis to retain in the final model(s).

3. Multiple Imputation for missing data

Multiple imputation for this study will be carried out within contributing datasets that have <65% missing data for the primary and select secondary predictors; blood glucose and blood leukocytes. Characteristics of participants with 10–64.99% missing data will be summarized and compared to those with ‘complete’ data (<10% missing data) to explore the nature of missingness and identify auxiliary variables that could later inform imputation. Comparison characteristics include sex, age, survival time (days), outcome, diagnosis, and TBM case status (definite, probable, possible, not-TBM). If there is no clear pattern of missingness, the data will be assumed missing at random and imputed. After imputation, the fraction of missing information (FMI) statistic will be estimated in the modeling step to ensure that the imputation model is well specified ^13^. It is not always reasonable to assume predictors missing ≥65% of data are missing at random. Therefore, we will attempt to determine the underlying mechanism of missingness and not impute for these predictors if the missingness cannot be accounted for by another variable with complete information or if the missing data appear to be missing not at random.

Auxiliary variables such as sex and age, which are typically predictive of most biological values, will be used to help inform imputation. Further auxiliary variables for each missing predictor will be selected based on biologic plausibility. For example, missing CSF glucose will be informed by blood glucose. After auxiliary variables are identified for each missing primary or secondary predictor, missing data will be imputed within each contributing dataset (i.e. not informed with data from other contributing datasets.

Multiple imputation will be carried out in R using the MICE package. Patients with TBM and other types of meningitis are typically acutely ill, therefore we are expecting skewed values for all the biologic metrics and will be utilizing the chained equations approach in the MICE package. We will impute 20 datasets for each missing variable.

4. Developing a Predictive Model

After that we will build a predictive algorithm via IPD meta-analysis using a logistic regression model for the diagnosis of TBM ^7^. The first step is to estimate the predictor–outcome associations from the available studies in order to assess heterogeneity in predictors across studies. Predictors that have homogenous predictor-outcome associations will be prioritized in model inclusion, but we will not exclude variables that have heterogenous predictor-outcome relationships across studies. All predictors will be included in a model with a stratified intercept for each study to underscore the baseline predictor-outcome value of each of the predictors in the different contributing datasets ^7^. We will also develop a model with a stratified intercept for each country (pooling datasets from the same country) for the purposes of external validation and implementation after the model is developed.

All the data collected from the systematic review will be used in the development of the clinical prediction tool. The objective of this step is to develop three different prediction models. The first model will be developed using logistic regression with backward stepwise variable selection (p-value threshold of 0.1) ^14, 15^. This is the modeling approach used in prior clinical prediction tools developed for TBM. The second model will be developed using the IPD meta-analysis framework with a stratified intercept for country ^7^. As discussed earlier, this approach is appropriate for these data as it encompasses information from multiple contributing datasets in the development of the tool. The final model will be developed using machine learning techniques such as classification and regression trees or random forest classifier analysis.

5. Testing the model for internal validity

The model(s) will be internally validated using the bootstrap and internal-external cross validation approaches. Bootstrap validation is the process for which observations from within each contributing dataset are sampled with replacement to go into the development of the model, the model development analyses are repeated, and then this model is internally validated in the original datasets ^16, 17^. This validation step will give information of optimism and overfitting. Internal-external cross validation approach is a multiple validation approach that accounts for multiple datasets by rotating which are used toward model development and validation ^7^. Each contributing dataset will be excluded from the available set, and the remainder will be used to develop the diagnostic model; the excluded study will then be used to validate the model externally. This process will be repeated with each study being omitted in turn, allowing the consistency of the developed model and its performance to be examined on multiple occasions.

Performance of the developed model(s) will be assessed using calibration and discrimination, metrics for model fit. Calibration is defined as the agreement between observed outcomes and predictions ^18^. We will use the ratio of predicted (expected) to observed outcomes, otherwise known as E/O, to assess model calibration. Ideally, the ratio should be close to 1, which represents a calibrated model ^7^. Calibration is also related to goodness-of-fit, which relates to the ability of a model to fit a given set of data ^18^. The Hosmer-Lemeshow goodness-of-fit test is often used to assess goodness-of-fit with binary outcome data, which can be graphically displayed in a calibration plot. Usually, patients are grouped by decile of predicted probability. A better calibrated model will have the average prediction value within each decile falling along a 45 degree line in the plot, where the true probability in each decile (y-axis) is equal to the average predicted probability for that group (x-axis) ^18^.

Discrimination is defined as the ability to generate predictions that discriminate between those with and those without the outcome (e.g. TBM vs not-TBM diagnosis) ^18^. We can assess discrimination using a receiver operating characteristic (ROC) curve, which plots the sensitivity (true positive rate) against 1 – specificity (false-positive rate) for consecutive cutoffs for the probability of an outcome (i.e. TBM diagnosis). For the bootstrap approach, ROC curves will be produced as an average for the models that are bootstrapped, and discrimination will be compared using the area under the curve (AUC) c-statistic. For the internal-external cross validation approach, discrimination will be estimated in each study that is excluded from development. The AUC can be interpreted as the probability that a patient with the outcome is given a higher probability of the outcome by the model than a randomly chosen patient without the outcome ^18^. The higher the AUC, represented by the curve closer to the upper left-hand corner (higher sensitivity and specificity), the better the model is at predicting the outcome, in this case TBM diagnosis. Furthermore, ROC curves are often used in diagnostic research to quantify the diagnostic value of a test over its whole range of possible cutoffs for classifying patients as positive vs. negative ^18^. The curves trend to the upper left corner when the distributions of predictions are more separate between those with and without the outcome.

Optimism or “overfitting” is where the model fits the data so well that it is not valid for new subjects, a key threat to internal validity that needs to be addressed throughout the internal validation process. Model performance statistics are generated and then optimism-corrected by taking the apparent performance and subtracting the optimism. The optimism for a particular statistic is calculated by repeating the model development in each bootstrap sample, calculating the performance in the bootstrap sample (where it will be optimistic), and then applying that model back to the original dataset (acting as a validation dataset). After repeating this process 100–200 times, the average of the differences between these model performance statistics is the estimate of optimism.

As discussed, high optimism is indicative of overfitting, which can be corrected via shrinkage. Shrinkage is defined as the regression of coefficients towards zero as a way to improve model performance ^18^. Although we do not anticipate that we will need to employ this method to correct for overfitting because we will have completed the data reduction step described in step 3 above, we will validate the necessity of the shrinkage approach when assessing model optimism.

In addition to assessing model calibration and discrimination, the Brier score will be used to assess overall model performance. The Brier score measures the accuracy of probabilistic predictions, which is a combination of both calibration and discrimination ^18, 19^. The Brier score calculates the squared differences between actual outcomes and predictions. For a model, the Brier score can range from 0% for a perfect model to 0.25 for a non-informative model. A Brier score will be generated from the model developed in the original dataset.

After the internal validation process, we will select model(s) that perform well and calculate the overall sensitivity, specificity, positive predictive value, and negative predictive to assess the accuracy of the model(s) in predicting TBM. Due to the high mortality among unstable TBM patients a 10% predictive probability will be used as a threshold of a positive case status ^20^. Any predictive probability equal to or above 10% will be sufficient to treat a patient for tuberculosis since the outcome of not treating TBM is almost always death ^20^.

6. Sensitivity analysis

We will perform the following sensitivity analyses to explore the contributions of risk of bias on the final model(s):

- Exclude studies that investigated all patients for TBM regardless of other CSF findings (co-infection). This is important for discerning which clinical characteristics are predictive of TBM or other meningitis-causing diseases.
- Develop the prediction model(s) with different TBM-case status groupings. Confirmed TBM and non-TBM cases will remain as such in model development with probable and possible cases shifting to either category. The model will be developed with the following case status groupings:
  - Confirmed TBM vs. probable, possible, and non-TBM
  - Confirmed and probable TBM vs. possible and non-TBM
  - Confirmed, probable, and possible TBM vs. non-TBM

We will compare the observed heterogeneity, clustering, predictor selection, and model performance between the models developed with the above case groupings. This is important for two reasons. First, shifting the threshold of inclusion or TBM “caseness” could provide further insight into the misclassification of cases as a result of poor diagnostic strategies for TBM. Second, including the information from probable and possible TBM cases in model development would result in a more applicable model for probable and possible cases, with the intention that the model could better predict TBM for these persons.

- Conduct a misclassification bias analysis. This step is important since there is no “gold-standard” diagnostic criteria for TBM diagnosis so there is likely misclassification bias. Many of the diagnostic methods used to ascertain TBM, such as TB culture in CSF, have known sensitivities and specificities that will be used towards reclassifying cases. Model(s) will be developed with the reclassified case statuses and compared to the model(s) developed with the original case classification.

We will also assess risk of bias for each included study using the QUADAS-2 tool ^11^. This tool comprises four domains: participant selection, predictor measurement, and outcome definition and measurement. Each domain is assessed in terms of risk of bias and are also assessed in terms of concerns regarding applicability. Signaling questions are included to help judge risk of bias.

## Registration

This review is registered with PROSPERO, number CRD42018110501.

## Presenting and reporting of results

We will report the results according to the Preferred Reporting Items for a Systematic Review and Meta-analysis of Individual Participant Data Statement (PRISMA-IPD) ^10^. This will include a flow diagram to summarise the study selection process and detail the reasons for exclusion of studies screened as full text. We will publish our search strategy and quality-scoring tool as supplementary documents. Quantitative data will be presented in evidence tables of individual studies as well as in summary tables. We plan to report on quality scores and risk of bias for each eligible study. This may be tabulated and accompanied by narrative summaries. A descriptive analysis of the strength of evidence assessment will be reported. The final prediction model(s), that is, the variable-selected model(s) with the highest area under the receiver operating characteristic curve (AUC), will be implemented in a Smart phone application and a Web-based calculator and graphically depicted using nomograms.

## Discussion

TBM is a serious public health concern with delayed diagnosis and treatment being important risk factors for poor outcome ^1^. At least 10 attempts have been made to develop clinical prediction models to aid the rapid diagnosis of TBM but none have been broadly successful. The aim of this project is to combine data from multiple sources to develop and internally validate a novel clinical prediction model, which will be made easily available as a smart phone application and a Web-based calculator. By combining data from multiple geographical locations and using advanced machine learning techniques it is hoped that we can develop a model that is broadly generalizable around the world. Further work will involve external validation of the model(s) and testing in randomised controlled trials.

## Ethics

No specific ethical approval has been sought for this systematic review. Authors who submit IPD will be asked to confirm that the dissemination of anonymised data was included in the original patient consent document.

## Data availability

### Underlying data

No data is associated with this article.

### Reporting guidelines

Figshare: PRISMA-P checklist for The diagnosis of tuberculous meningitis in adults and adolescents: protocol for a systematic review and individual patient data meta-analysis to inform a multivariable prediction model, https://doi.org/10.6084/m9.figshare.7628639.v1

Data are available under the terms of the Creative Commons Attribution 4.0 International license (CC-BY 4.0).
