# Supplementary material for: The diagnosis of tuberculous meningitis in adults and adolescents: protocol for a systematic review and individual patient data meta-analysis to inform a multivariable prediction model
Source: Wellcome Open Res. 2021 Jan 4;4:19. Originally published 2019 Jan 31. [Version 3] doi: 10.12688/wellcomeopenres.15056.3 (PMC7863992; doi:10.12688/wellcomeopenres.15056.3)
Supplement: Peer review history

# Peer review: Reviewer response for version 3

**DOI:** 10.21956/wellcomeopenres.18198.r41935

**Published:** 2021-02-04

**Authors:** Kym I.E. Snell

**Affiliations:** 1 Centre for Prognosis Research, School of Medicine, Keele University, Keele, UK

I thank the authors for taking my previous comments on board and addressing many of my concerns. The method section is vastly improved in this latest version. I hope that the authors will consider using risk of bias tools and reporting guidelines developed specifically for diagnostic (and prognostic) model studies such as PROBAST and TRIPOD. An extension for TRIPOD covering clustered data (including IPDMAs) is also underway and likely to be published prior to this study being written up.

 I wish the group the best of luck with their research.

Is the study design appropriate for the research question?

Yes

Is the rationale for, and objectives of, the study clearly described?

Yes

Are sufficient details of the methods provided to allow replication by others?

Partly

Are the datasets clearly presented in a useable and accessible format?

Not applicable

Reviewer Expertise:

Biostatistics, prediction modelling and IPD-MA

I confirm that I have read this submission and believe that I have an appropriate level of expertise to confirm that it is of an acceptable scientific standard.

# Peer review: Reviewer response for version 2

**DOI:** 10.21956/wellcomeopenres.16866.r36627

**Published:** 2019-10-25

**Authors:** Kym I.E. Snell

**Affiliations:** 1 Centre for Prognosis Research, School of Medicine, Keele University, Keele, UK

The authors have addressed some of my previous comments. However, there are several points that I have to still query.

 Firstly, I think there has been some confusion in the use of the terms ‘case-mix variation’ and heterogeneity. As the project includes an IPD-MA, I was referring to differences in case-mix across studies and heterogeneity in performance across studies in line with text in the introduction. Therefore, some of the changes to the protocol are rather confusing.

 If the authors intend to look at heterogeneity in predictor effects, their approach (a stratified intercept model with fixed effect for the predictor effect) will not allow them to investigate this as the predictor effect is fixed. If heterogeneity in predictor effects is of interest, then I would suggest placing a random effect on the predictor effect allowing the slope to change by study and reporting estimates of heterogeneity. If this is not of interest, then please remove mention of investigating heterogeneity in predictor effects.

 It is not clear what is meant by ‘We will also use this method to assess predictor heterogeneity by HIV status, WHO region, and TB burden within each country’? Do you mean evaluate predictors within subgroups of these factors, fit interactions between predictors and these effects, or something else?

 I’m also rather confused by the suggestion of classification and regression trees, supervised and unsupervised ML, LCA in relation to case-mix variation. These are just different statistical techniques that can be used to investigate clustering of individuals (by characteristics) and doesn’t explicitly say how different studies will be used or what the objective is.

 The methods for model development and validation are still vague and suggest to me that the data from multiple studies will be combined and treated as a single dataset rather than as an IPD-MA (accounting for clustering by study). IPD-MA can be very challenging, however there is a lot of guidance on how to conduct them, particularly by T. Debray and R. Riley. Sadly, much of the guidance has either not been considered or been ignored. If using regression modelling, clustering by study could be accounted for by using a random intercept or stratified intercept. It is then also important to decide how the model would be implemented i.e. what intercept would be used for validation in a new study or for new patients in practice (Debray *et al.*, 2013 ^1^)? I am not aware of how clustering can be accounted for when using methods such as CART.

 I have some concern about the decision not to impute for missing data, especially given the list of 28 predictors of interest and no minimum sample size that they aim to achieve. In my experience, expecting to receive datasets with 28 predictors of interest is incredibly optimistic. It is more likely that both systematically missing data and partially missing data will be a challenge. If every dataset was missing just one of those variables, there would be no data left to use. It often ends up being a compromise between the number of predictors (and which ones are) considered and the number of datasets included. If then also not imputing for partially missing data, further data is lost and predictor effects may be biased (e.g. only severe cases get certain tests). This could affect your target population and result in models that are not applicable to the population of interest.

 Regarding the decision to develop the model in only confirmed TBM and non-TBM patients, I would think that the target population would include probable and possible TBM and therefore there would be an applicability issue if the model was developed using an unrepresentative subgroup of patients (Moons *et al.*, 2019 ^2^). These probable and possible cases are also often the individuals in which a prediction model has the most potential to be useful. Other options to consider are to include them with one or the other of confirmed TBM and non-TBM so as not to exclude them. Sensitivity may be to change which outcome they’re grouped with.

 Finally the description of bootstrap validation is not correct. Optimism is not a statistic as such but can be estimated for each performance statistic of interest. The 'optimism-corrected' statistics are then calculated as apparent minus optimism. The optimism for a particular statistic is calculated by repeating the model development in each bootstrap sample, calculating the performance in the bootstrap sample (where it will be optimistic) and also applying that model back to the original dataset (acting as a validation dataset). Repeat lots of times and the average of the differences between these is the estimate of optimism. Estimating optimism by bootstrapping is sufficient, no need to calculate for other validation approaches like IECV.

Is the study design appropriate for the research question?

Yes

Is the rationale for, and objectives of, the study clearly described?

Yes

Are sufficient details of the methods provided to allow replication by others?

Partly

Are the datasets clearly presented in a useable and accessible format?

Not applicable

Reviewer Expertise:

Biostatistics, prediction modelling and IPD-MA

I confirm that I have read this submission and believe that I have an appropriate level of expertise to confirm that it is of an acceptable scientific standard, however I have significant reservations, as outlined above.

## References

[1] : A framework for developing, implementing, and evaluating clinical prediction models in an individual participant data meta-analysis. *Stat Med*.2013;32(18) : 10.1002/sim.5732 3158-80 10.1002/sim.5732 23307585

[2] : PROBAST: A Tool to Assess Risk of Bias and Applicability of Prediction Model Studies: Explanation and Elaboration. *Annals of Internal Medicine*.2019;170(1) : 10.7326/M18-1377 10.7326/M18-1377 30596876

# Peer review: Reviewer response for version 1

**DOI:** 10.21956/wellcomeopenres.16426.r34913

**Published:** 2019-03-08

**Authors:** Kym I.E. Snell

**Affiliations:** 1 Centre for Prognosis Research, School of Medicine, Keele University, Keele, UK

This is a clear and well-written protocol for a systematic review, collection of IPD and IPD meta-analysis for a new diagnosis model for TBM. I think the aim of the study and details relating to the systematic review part are clear, however I have a few comments/questions for clarity and reproducibility, mostly regarding what happens once IPD has been collected.

1. In the introduction the authors mention that case-mix can affect the predictive performance of a model and that big datasets can be used to examine the heterogeneity and improve the predictive performance. However, I don’t think they really address this issue in the methods or say how they will use the IPD to try improve the performance. Heterogeneity in performance if the predictor effects are consistent suggests differences in case-mix that are not being captured by the predictors in the model. Unless additional variables that are thought to improve the model are included, how will this be addressed? Will the authors consider recalibrating the baseline risk to different populations for example?
2. For the risk of bias assessment, I suggest using items from PROBAST too (excluding the analysis domain) which has recently been published and relates to prediction modelling studies (Wolff *et al.*, 2019 ^1^).
3. Have the authors considered how much data they would need to acquire to develop new prediction models for TBM e.g. any sample size calculations based on likely event rate and expected number of candidate predictors for consideration in the models, as a target to aim for?
4. In my experience, one of the biggest difficulties with IPD-MA like these is how different studies record different combinations of variables. Therefore, combining studies for model development can be very difficult and it may be necessary to prioritise certain variables (or combinations of variables) and use a subset of studies with those variables, hence my previous comment regarding sample size. Have the authors considered which variables are of particular interest and what they will do if these are not recorded in individual studies? How will IPD be selected for developing new models as it is unlikely to all be used?
5. How will missing data be handled? If imputing, will this be done within or across datasets?
6. It’s not clear if one model will be developed or multiple models (using each of the different modelling approaches). If aiming for a single model, how will it be selected?
7. Bottom of page 5: I’m not sure what is meant by “Model development will initially be carried out using participants with either definite TBM or definitely not TBM. The model will then be applied to participants with possible TBM.” Can the authors please clarify? Do they mean that possible TBM will be included in the definition of TBM?
8. I’m also not sure what is meant by “the training set will be calibrated to optimise the model coefficients for best predictive accuracy using AUC-ROC score” (Data synthesis, part 2)? By definition, the model will be calibrated to the development data and is therefore optimised to the data, which can lead to overfitting.
9. Will clustering by dataset be accounted for in the model development e.g. using a random intercept?
10. Will calibration of the model be assessed? This is also likely to be heterogeneous in different populations and therefore may need tailoring to different populations. In contrast, the AUC depends on the case-mix and will be lower in more homogeneous populations which doesn’t mean the model doesn’t work well.
11. I don’t see the point in splitting each dataset for development and validation, especially when some studies are likely to be small (min. sample size of 10 so even fewer events). The internal-external cross-validation is a better approach as it still retains the external validation element and will help evaluate the heterogeneity in performance across datasets.
12. Have the authors considered the potential for optimism in model development, particularly if they have few events and small sample size overall? Will they consider shrinking the coefficients (in a regression modelling approach) to correct for optimism?
13. Data synthesis, part 3: What threshold will be selected to calculate measures of diagnostic test accuracy – will this be based on a predicted probability and pre-specified to avoid bias in using ‘optimal’ thresholds? I would also suggest evaluating calibration and discrimination as part of the internal validation.
14. I would suggest reporting according to the TRIPOD guidelines for the multivariable modelling (Collins *et al.*, 2015 ^2^).
15. I would caution against simply developing smart phone apps and web-based calculators unless the model demonstrates good predictive ability. Ideally it should be externally validated first before considering it as a tool for use in practice.

Is the study design appropriate for the research question?

Yes

Is the rationale for, and objectives of, the study clearly described?

Yes

Are sufficient details of the methods provided to allow replication by others?

Partly

Are the datasets clearly presented in a useable and accessible format?

Not applicable

Reviewer Expertise:

Biostatistics, prediction modelling and IPD-MA

I confirm that I have read this submission and believe that I have an appropriate level of expertise to confirm that it is of an acceptable scientific standard, however I have significant reservations, as outlined above.

## References

[1] : PROBAST: A Tool to Assess the Risk of Bias and Applicability of Prediction Model Studies. *Ann Intern Med*.2019;170(1) : 10.7326/M18-1376 51-58 10.7326/M18-1376 30596875

[2] : Transparent reporting of a multivariable prediction model for individual prognosis or diagnosis (TRIPOD): the TRIPOD statement. *BMJ*.2015;350: 10.1136/bmj.g7594 g7594 10.1136/bmj.g7594 25569120

# Response

**Published:** 2019-08-21

**Authors:** Tom Boyles

**Affiliations:** Helen Joseph Hospital, South Africa

We would like to thank Professor Snell for her very helpful comments.

Responses drafted by Anna Stadelman.

READ ME: **Comments from Professor Snell are in bold-type font**. My responses are below each comment.

                 

**This is a clear and well-written protocol for a systematic review, collection of IPD and IPD meta-analysis for a new diagnosis model for TBM. I think the aim of the study and details relating to the systematic review part are clear, however I have a few comments/questions for clarity and reproducibility, mostly regarding what happens once IPD has been collected.**

**1. In the introduction the authors mention that case-mix can affect the predictive performance of a model and that big datasets can be used to examine the heterogeneity and improve the predictive performance. However, I don’t think they really address this issue in the methods or say how they will use the IPD to try [to] improve the performance. Heterogeneity in performance if the predictor effects are consistent suggests differences in case-mix that are not being captured by the predictors in the model. Unless additional variables that are thought to improve the model are included, how will this be addressed? Will the authors consider recalibrating the baseline risk to different populations for example?**

The objective of assessing heterogeneity will be to ascertain case-mix variation among TBM cases and non-cases to inform model development. To begin, the contributing datasets will be reviewed for sample size, available predictors, and data completeness to inform the selection of a modelling approach. A descriptive analysis will be undertaken to understand similarities and differences between the contributing datasets. Participant characteristics and clinical features will be summarized for each contributing dataset and compared across datasets using chi-square, t-tests, or non-parametric methods as warranted. Then, we will formally evaluate case-mix variation and predictor heterogeneity via IPD meta-analysis using a logistic regression model with stratified intercepts for each study (99). Each TBM predictor will be rotated into the model individually to underscore the baseline predictive value of each in the different contributing datasets. We will also use this method to assess predictor heterogeneity by HIV status, WHO region, and TB burden within each country. For both the informal (descriptive statistics) and formal (IPD meta-analysis) assessment of heterogeneity, predictor estimates and their uncertainty intervals will be used to determine relative significance as opposed to p-values. Uncertainty intervals for each predictor will indicate how reliable the predictor is in terms of its prediction value. Furthermore, looking at p-values will only assess statistical significance, which may not be clinically meaningful.

Subsequently, we will employ methods of model development that take into account the heterogeneity observed in the IPD meta-analysis. These methods include, but are not limited to, classification and regression trees, supervised and unsupervised machine learning, Latent component analysis, etc. There may be other sources of heterogeneity that become evident during model development which will also be included in the development of the clinical prediction rule.  

**2. For the risk of bias assessment, I suggest using items from PROBAST too (excluding the analysis domain) which has recently been published and relates to prediction modelling studies (Wolff *et al.*, 2019** **^1^** **).**  

Thanks for the recommendation.

**3. Have the authors considered how much data they would need to acquire to develop new prediction models for TBM e.g. any sample size calculations based on likely event rate and expected number of candidate predictors for consideration in the models, as a target to aim for?**

Sample size is difficult to calculate in the context of developing a prediction model. However, the size of the development dataset and number of predictors in the final model have an impact on the statistical power to detect a difference in TBM case vs. non-TBM case. The greater the number of individual participants the more information we have to inform the development of the model, specifically the parameterization of the predictors and variability explained. More data (i.e. individual participants) better optimizes the individual predictors and has a better chance of capturing the variability in TBM case presentations. Ultimately, we will make every effort to acquire as many datasets as possible and limit the number of predictors to the ones that explain the most variability in TBM diagnosis.

**4. In my experience, one of the biggest difficulties with IPD-MA like these is how different studies record different combinations of variables. Therefore, combining studies for model development can be very difficult and it may be necessary to prioritise certain variables (or combinations of variables) and use a subset of studies with those variables, hence my previous comment regarding sample size. Have the authors considered which variables are of particular interest and what they will do if these are not recorded in individual studies? How will IPD be selected for developing new models as it is unlikely to all be used?**

We have included in the table which variables are of interest in the development of the model(s) (marked with an *). We consider these variables to be the most important for model development and inclusion of data into model development will be contingent on the representation of these variables in the individual contributing datasets. We will conduct a sensitivity analysis with all the individual contributing datasets, regardless of variable inclusion, so as to assess any bias introduced into the model by excluding certain datasets. 

**5.** **How will missing data be handled? If imputing, will this be done within or across datasets?**

We will not impute any missing data. We will request all the diagnostic data available from investigators and any missingness on an individual level may ultimately end up excluding that particular individual from model development.   

**6. It’s not clear if one model will be developed or multiple models (using each of the different modelling approaches). If aiming for a single model, how will it be selected?**

We will create multiple models with the development dataset and compare the fit across models via bootstrap, k-fold cross validation, and internal-external cross-validation.

**7. Bottom of page 5: I’m not sure what is meant by “Model development will initially be carried out using participants with either definite TBM or definitely not TBM. The model will then be applied to participants with possible TBM.” Can the authors please clarify? Do they mean that possible TBM will be included in the definition of TBM?**

The model(s) will be developed with confirmed TBM and non-TBM cases, and we will test the model(s) on probable and possible TBM cases as part of the sensitivity analysis.

**8. I’m also not sure what is meant by “the training set will be calibrated to optimise the model coefficients for best predictive accuracy using AUC-ROC score” (Data synthesis, part 2)? By definition, the model will be calibrated to the development data and is therefore optimised to the data, which can lead to overfitting.**

Sorry for the confusion. Suggested revision in Version 2.0 of the protocol.

**9. Will clustering by dataset be accounted for in the model development e.g. using a random intercept?**

Yes, this will be the aim of the IPD meta-analysis. Each predictor of interest will be rotated into a model predicting TBM that has a random intercept for each contributing dataset. The aim of this will be to ascertain heterogeneity in predictor strength, which will be accounted for in the final model(s). However, the overall aim is to develop a model(s) that accounts for heterogeneity by region, HIV status, and other known causes of heterogeneity in TBM cases. Therefore, we are hoping that including these predictors in the final model(s) will account for most of the variation in predictor strength that may be introduced by individual contributing datasets.

**10. Will calibration of the model be assessed? This is also likely to be heterogeneous in different populations and therefore may need tailoring to different populations. In contrast, the AUC depends on the case-mix and will be lower in more homogeneous populations which doesn’t mean the model doesn’t work well.**

Yes, model(s) calibration will be assessed and you bring up important points about the metrics for calibration and discrimination.  

**11. I don’t see the point in splitting each dataset for development and validation, especially when some studies are likely to be small (min. sample size of 10 so even fewer events). The internal-external cross-validation is a better approach as it still retains the external validation element and will help evaluate the heterogeneity in performance across datasets.**

Agreed. We will revise our internal validation approach to include bootstrap, cross-validation (k-fold), and internal-external validation. Bootstrap validation tells us more about the validity of predictor variable selection in algorithm development, which is useful for assessing how well our predictors assess TBM diagnosis within different samples. Simulations have demonstrated that bootstrap is the best approach to internal validation as it appropriately reflects all sources of model uncertainty, especially in predictor variable selection (113). We will then utilize a k-fold cross-validation approach to assess the validation of the model approach and accuracy of model fit. The resulting c-statistic will convey overall model optimism and accuracy of model fit. The predictive model(s) will be further validated using ‘internal-external cross-validation’, which is a multiple validation approach that accounts for multiple studies by rotating which are used toward model development and validation (99).

**12. Have the authors considered the potential for optimism in model development, particularly if they have few events and small sample size overall? Will they consider shrinking the coefficients (in a regression modelling approach) to correct for optimism?**

High optimism, indicative of overfitting, can be corrected via shrinkage and we will consider this approach if overfitting is evident. However, we do not anticipate that we will encounter overfitting due to the data reduction step described in Version 2.0 of the protocol.

**13. Data synthesis, part 3: What threshold will be selected to calculate measures of diagnostic test accuracy – will this be based on a predicted probability and pre-specified to avoid bias in using ‘optimal’ thresholds? I would also suggest evaluating calibration and discrimination as part of the internal validation.**

We will assess optimism, calibration, and discrimination as part of the internal validation approach and have discussed this process further in Version 2.0 of the protocol. As for determining a pre-specified predictive threshold for defining TBM versus not, there is little information in the literature to inform an appropriate cutoff for TBM. Prior prediction models have used ROC curve to determine an optimal cutoff. Furthermore, this is the first study to include data from different populations world-wide. As such it is difficult to pre-specify the optimal predictive cutoff.

**14. I would suggest reporting according to the TRIPOD guidelines for the multivariable modelling (Collins *et al.*, 2015** **^2^** **).**

Great! Thanks for the recommendation.

**15. I would caution against simply developing smart phone apps and web-based calculators unless the model demonstrates good predictive ability. Ideally it should be externally validated first before considering it as a tool for use in practice.**

Absolutely agree. Developing an application and/or website calculator is our end goal, but the step to getting there includes further external validation.

**References**

1. Wolff RF, Moons KGM, Riley RD, Whiting PF, Westwood M, Collins GS, Reitsma JB, Kleijnen J, Mallett S, PROBAST Group†: PROBAST: A Tool to Assess the Risk of Bias and Applicability of Prediction Model Studies. *Ann Intern Med*. 2019; **170** (1): 51-58 PubMed Abstract | Publisher Full Text 

2. Collins GS, Reitsma JB, Altman DG, Moons KG: Transparent reporting of a multivariable prediction model for individual prognosis or diagnosis (TRIPOD): the TRIPOD statement. *BMJ*. 2015; **350**: g7594 PubMed Abstract | Publisher Full Text

# Peer review: Reviewer response for version 1

**DOI:** 10.21956/wellcomeopenres.16426.r34749

**Published:** 2019-02-05

**Authors:** Ravindra Kumar Garg

**Affiliations:** 1 Department of Neurology, King George's Medical University, Lucknow, Uttar Pradesh, India

I read with interest the protocol that aims to identify and validate a set of clinical predictors that accurately identify patients with definite tuberculous meningitis and absence of tuberculous meningitis. Conventionally, microscopy for acid-fast bacilli, commercial nucleic acid amplification test for *Mycobacterium tuberculosis* or mycobacterial culture of cerebrospinal fluid, are the tests that are used to bacteriologically confirm the diagnosis of tuberculous meningitis.

 In developing countries and countries with a very high tuberculosis burden, tuberculous meningitis is encountered very frequently. Tuberculous meningitis is the commonest CNS infection seen in Neurology and Medicine indoors. Facing resource constrains, we always have to rely on clinical, imaging and cerebrospinal fluid parameters. Despite constrains, we are able to make reliable diagnosis of tuberculous meningitis most of the time. Our classical teaching points, to diagnose tuberculous meningitis, are often accurate. With a clinical diagnosis of meningitis along with characteristic cerebrospinal fluid findings help in making reasonable and prompt diagnosis enabling to start antituberculosis treatment with confidence. Raised cerebrospinal fluid lymphocyte count and markedly raised protein are characteristically seen in tuberculous meningitis.

 Certain clinical signs are very specific to tuberculous meningitis. For example, sixth nerve involvement and vision loss in points towards a basal meningeal involvement and tuberculous meningitis. Other cranial nerve involvements are very infrequent. In patients with multiple cranial nerve palsies, fungal infection and a malignancy are more likely possibilities. As per observation, headache and fever are often not dominant features, and they are never presenting features. Similarly, neck rigidity may not be present in many patients. In cryptococcal meningitis, severe and dominant headache may be a presenting feature. Presence of extrapyramidal movements is a rare manifestation of tuberculous meningitis in adults. Extrapyramidal movements are more frequent in children.

 Computed tomographic findings, if present, are quite characteristic of tuberculous meningitis. Basal exudates along with hydrocephalus with or without tuberculoma and periventricular infarcts indicates tuberculous meningitis and differential diagnosis option are then limited. A search for spinal cord involvement, we believe, if present, add to the diagnostic accuracy.  A combination of optochiasmatic arachnoiditis and spinal  lumbo-sacral arachnoiditis, in my opinion, is probably as accurate as bacteriological confirmation. Demonstration of paradoxical reaction, if present, also helps us in substantiating the reliable diagnosis of tuberculous meningitis.

 Tuberculous meningitis, frequently, is a manifestation of more disseminated tuberculosis. Search for other sites of involvement often help us establishing clinical diagnosis. For example, ordinary X-ray chest shows additional pulmonary involvement. Many cases surprisingly show asymptomatic military tuberculosis. Lymph adenopathy and spinal vertebral tuberculosis are also seen in many cases.

 Diagnostic caution is exercised in elderly patients and HIV infected patients. In these two groups, there are high chances of alternative diagnosis. We routinely perform tests with India ink preparation and detection of malignant cells. Still, distinctive features of tuberculous meningitis help in diagnosis of tuberculous meningitis in these two populations as well. Aspergillosis has a more aggressive course and large vessel involvement is more common. In tuberculous meningitis infarcts are usually small and periventricular. 

 Another issue that need to be addressed is diagnosis of drug-resistant tuberculous meningitis. XpertMTB/RIF test, which is now readily available, start discovering drug-resistant tuberculous meningitis in increasing number.  This is not surprising because India harbors the major portion of global drug-resistant tuberculosis problems. This issue also needs to be given due emphasis.

 I greatly appreciate the investigators efforts to evolve a predictive logistic model to accurately diagnose definite tuberculous meningitis. There are certain points that I highlighted that need to be re-looked and can be incorporated in this protocol.

Is the study design appropriate for the research question?

Yes

Is the rationale for, and objectives of, the study clearly described?

Yes

Are sufficient details of the methods provided to allow replication by others?

Yes

Are the datasets clearly presented in a useable and accessible format?

Not applicable

Reviewer Expertise:

CNS tuberculosis and other CNS infections

I confirm that I have read this submission and believe that I have an appropriate level of expertise to confirm that it is of an acceptable scientific standard.
